# Emerging concepts regarding pro- and anti tumor properties of B cells in tumor immunity

**DOI:** 10.3389/fimmu.2022.881427

**Published:** 2022-07-28

**Authors:** You Qin, Furong Lu, Kexing Lyu, Alfred E. Chang, Qiao Li

**Affiliations:** ^1^ Cancer Center, Union Hospital, Tongji Medical College, Huazhong University of Science and Technology, Wuhan, China; ^2^ Rogel Cancer Center, University of Michigan, Ann Arbor, MI, United States; ^3^ Department of Integrated Traditional Chinese and Western Medicine, Union Hospital, Tongji Medical College, Huazhong University of Science and Technology, Wuhan, China; ^4^ Department of Molecular and Integrative Physiology, University of Michigan Medical School, Ann Arbor, MI, United States; ^5^ Otorhinolaryngology Hospital, The First Affiliated Hospital, Sun Yat-sen University, Guangzhou, China

**Keywords:** B-lymphocytes, tumor-infiltrating lymphocytes, tertiary lymphoid structures, humoral immunity, immunotherapy, tumor microenvironment, prognosis

## Abstract

Controversial views regarding the roles of B cells in tumor immunity have existed for several decades. However, more recent studies have focused on its positive properties in antitumor immunity. Many studies have demonstrated a close association of the higher density of intratumoral B cells with favorable outcomes in cancer patients. B cells can interact with T cells as well as follicular dendritic cells within tertiary lymphoid structures, where they undergo a series of biological events, including clonal expansion, somatic hypermutation, class switching, and tumor-specific antibody production, which may trigger antitumor humoral responses. After activation, B cells can function as effector cells *via* direct tumor-killing, antigen-presenting activity, and production of tumor-specific antibodies. At the other extreme, B cells can obtain inhibitory functions by relevant stimuli, converting to regulatory B cells, which serve as an immunosuppressive arm to tumor immunity. Here we summarize our current understanding of the bipolar properties of B cells within the tumor immune microenvironment and propose potential B cell-based immunotherapeutic strategies, which may help promote cancer immunotherapy.

## Introduction

The host immune system has proven to be a powerful tool functioning in cancer progression control (1). Owing to the direct tumor-killing effect of CD8^+^ cytotoxic T cells, T cell responses have become the driving force in the recent therapeutic advances in cancer management (2, 3). For example, chimeric antigen receptor-T (CAR-T) cell therapy has paved a new way in cancer treatment (2). CAR-T cell immunotherapy has achieved impressive strides in hematopoietic malignancies while exhibiting limited activity against solid tumors because of the poor infiltration and persistence of CAR-T cells and immunosuppressive microenvironment (2). Immune checkpoint blockade (ICB) has also been a major new approach to cancer immunotherapy which has been shown to enhance antitumor T cell immunity as its major mode of action (3). However, response rates of ICB remain relatively low, ranging from 15% to 40% based on cancer types (3). B cells are another major subpopulation of lymphocytes that mediate the humoral immunity of the adaptive immune system. However, they are often overlooked in the field of cancer immunotherapy, likely due to the general notion that humoral and cellular immunity tend to work in opposing fashions (4). Actually, B cells occupy a central position in forming the tumor immune microenvironment (4), and deserve far more attention to its diverse immune functions, both positive and negative.

Both antitumor and tumor-promoting functions of B cells have been reported in tumor immunity and immunotherapy (4). Evidence accumulating in the late 1990s facilitated a widespread acceptance of protumor functions mediated by B cells (5). However, more recent findings have shown that B cells employ a protective rather than a detrimental property in malignant diseases (6–13). Despite limited evidence regarding the negative prognostic value of tumor-infiltrating B lymphocytes (TIL-Bs), more recent cohort studies indicate the close association of elevated intratumoral B cells with prolonged survival of cancer patients (14). In this review, we highlight the latest findings of B cell biology in the tumor microenvironment (TME) as well as promising strategies targeting B cells, which may help promote cancer immunotherapy.

## TIL-B recruitment, location, and subsets

**Figure 1:**
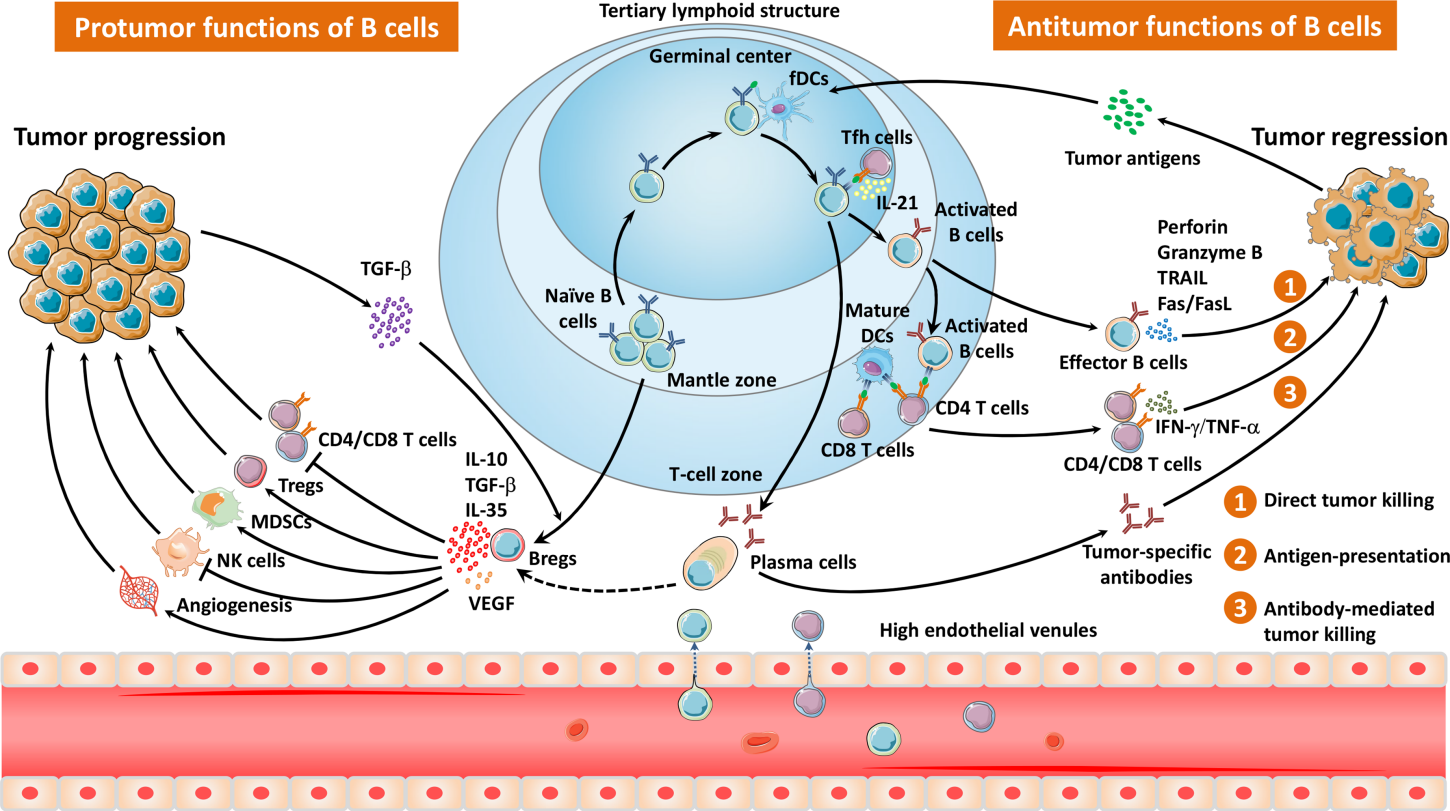
The migration pathways and diverse properties of TLS- and non-TLS-associated B cells in the TME. Naïve B cells are recruited and infiltrate into the TME *via* HEVs, mainly locating in the mantle zone of TLSs. After exposure to tumor antigens within the GCs, B cells interact with follicular helper T cells as well as follicular dendritic cells, and undergo a series of biological events, including clonal expansion, somatic hypermutation, class switching, and tumor-specific antibody production. Effector B cells or plasma cells elicit antitumor responses *via* direct tumor killing, antigen presentation, and antibody-mediated tumor cell lysis. In contrast, Bregs impair antitumor immunity through immunosuppressive cytokines, which inhibit the activity of T cells as well as NK cells, and induce Tregs, MDSCs, and angiogenesis.

Immune cells are recruited and infiltrate into tumor sites, forming a tumor immune microenvironment, where B cells comprise a considerable part of tumor-infiltrating lymphocytes (TILs). The recruitment of TIL-Bs into tumors is dependent on high endothelial venules (HEVs) (15), chemokines (16), and other immune cells (17). HEVs are postcapillary venules composed of plump endothelial cells, which allow entry of B lymphocytes into tertiary lymphoid structures (TLSs) (**Figure 1**) (15). In addition to HEVs, chemokines secreted by tumor cells and other immune components, such as CXCL2, CXCL20, and CXCL13, promote the influx of B cells into the tumor (16). Furthermore, CXCL13^+^CD103^+^CD8^+^ tumor-infiltrating T cells (TIL-Ts) were reported to correlate with B-cell recruitment in six cohorts of human tumors (17). In short, HEVs provide a channel for B cells to enter tumor niches, while T cells and multiple chemokines provide a driving force.

The majority of TIL-Bs exhibit a diffuse pattern in the peritumoral zone or within tumors (18). However, they also aggregate as small unorganized clusters or mature TLSs (6–8), which can be localized in the tumor center and, more frequently, in tumor margins or stroma (19).

TLSs are ectopic lymphoid tissues within the tumor which were initially defined as tumor-localized ectopic lymph node-like structures by Mulé and his colleagues as early as in the 1970s (20). Mature TLSs contain one or more germinal centers (GCs) with CD23^+^ germinal B cells as well as CD21^+^ follicular dendritic cells (fDCs) (6, 7). GCs are surrounded by dispersed T cells, mature dendritic cells (DCs), and plasma cells as well as HEVs (6). Intriguingly, the presence or absence of presumed TLSs can be accurately predicted by a 12-chemokine gene expression signature (21). Tumor TLSs support local adaptive immune responses *via* providing interaction between tumor antigen-specific lymphocytes and antigen-presenting cells (**Figure 1**). TLS-resided B cells may undergo a series of biological events, including clonal expansion, somatic hypermutation, class switching, and tumor-specific antibody production, thus triggering antitumor humoral responses (**Figure 1**) (19). More recently, Meylan *et al.* reported that all steps of B cell maturation occur in TLS until plasma cell formation followed by migration in the tumor (13). Interactions between B cells and T cells in the TLSs tend to be essential to improve the T cell-dependent antitumor immunity (19). Indeed, the presence of TLSs in TME has been demonstrated to be associated with favorable outcomes in several kinds of solid tumors (22–32).

Whether B cells drive or impair tumor growth might largely depend on the complicated and dynamic TME as well as certain B-cell subsets (**Figure 1**) (7). CD20^+^CD38^−^CD27^−^IgD^+^ naïve B cells enter tumors, usually localizing at the mantle zone. Once entering the GCs, most of B cells display the proliferation marker Ki67, activation-induced deaminase (AID) as well as transcription factor BCL-6 (6–8, 19). CD20^+^CD27^+^IgD^+^ non-switched memory B cells and CD20^+^CD27^+^IgD^−^ switched memory B cells accumulate at the interface between the mantle zone and the GCs (7). Plasma cells (CD20^−^CD38^hi^/CD138^+^) distribute in the periphery of follicle, tumor stromal, and fibrotic areas. Interestingly, Bruno et al. identified two phenotypes of TIL-Bs in non-small-cell lung cancer (NSCLC) as CD20^+^CD69^+^CD27^+^CD21^+^ activated TIL-Bs and CD20^+^CD69^+^CD27^-^CD21^-^ exhausted TIL-Bs, which were related to an effector T cell phenotype (CD4^+^IFNγ^+^) or a regulatory T cell (Treg) phenotype (CD4^+^FoxP3^+^), respectively (33). Recently, a distinct TIL-B subset (IgG4^+^CD49b^+^CD73^+^) was reported to express proangiogenic cytokines in patients with esophageal cancer and melanoma (34). Another study by Lu et al. described a novel ICOSL^+^ B cell subset emerging after chemotherapy with the capacity of reversing the chemoresistance of breast cancer patients (9). An overview of B cells in the TME is shown in **Figure 1**.

## Prognostic value of TIL-Bs in cancer patients

Given the convenience of detecting B-cell density using immunohistochemistry or flow cytometry, TIL-B and its subsets have become a practical and popular prognostic factor for solid tumors. Many cohort studies have uncovered a close association of TIL-Bs with clinical prognosis in cancer patients (14). Intriguingly, there exist considerable controversies over the prognostic impacts of TIL-Bs in different cancer types or subtypes (14). The high density of TIL-Bs has been identified as a biomarker indicating either favorable or unfavorable outcomes.

Growing evidence has revealed that TIL-B is a positive prognostic indicator in various types of human cancers. For example, Garaud et al. demonstrated the correlation between higher TIL-B densities and superior outcomes in HER2^+^ and triple-negative breast cancer patients with a 10-year median follow-up (35). Similarly, B cells in TLSs were detected to have a strong prognostic value of better outcomes in early and advanced NSCLC patients (36). In melanoma, tumoral CD20^+^ B cells were correlated with improved survival (8). Likewise, a TCGA data analysis implicated prolonged survival in melanoma patients with B cell-lineage-high tumors (7). A cohort study of oropharyngeal cancer demonstrated that abundant intratumoral CD20^+^ B cells exhibited longer overall survival (37). Additionally, a high density of TIL-Bs has been reported to be a favorable prognostic marker for patients of digestive system cancers, such as esophageal cancer (38), gastric cancer (39), colorectal cancer (40), pancreatic cancer (18), and hepatocellular carcinoma (HCC) (41). Furthermore, elevated intratumoral B cells have been identified as a positive prognostic index for genitourinary cancers, including ovarian cancer (42) and renal cell carcinoma (43). Recently, we carried out a meta-analysis and reported that TIL-B is a favorable prognostic biomarker in breast cancer (44).

By contrast, a limited number of publications have implicated that TIL-Bs were associated with a worse prognosis in certain cancer types. For instance, increased intratumoral B cells, especially TLS-locating CD20^+^ B cells, had a positive association with poor survival in lung adenocarcinoma patients (45). Additionally, a higher density of TIL-Bs present in melanoma tumors was correlated with reduced overall survival (46). Among the low-risk patients of oropharyngeal and hypopharyngeal cancers, low CD20^+^ B cell numbers implied notably better survival (47). Also, the presence of TIL-Bs was correlated with shorter survival and enhanced tumor aggressiveness in HCC (48). For ovarian cancer, the median overall survival was remarkably longer in patients with low B-cell gene expression than in those with high expression (49). Moreover, colorectal patients with positive TIL-Bs showed poor outcomes, which should be taken carefully due to the very few TIL-B positive cases (50). Sjöberg et al. reported that the B cell-high subgroup of renal cell carcinoma patients displayed markedly shorter survival, which was validated by analyses of other gene expression datasets (51).

**Figure 2:**
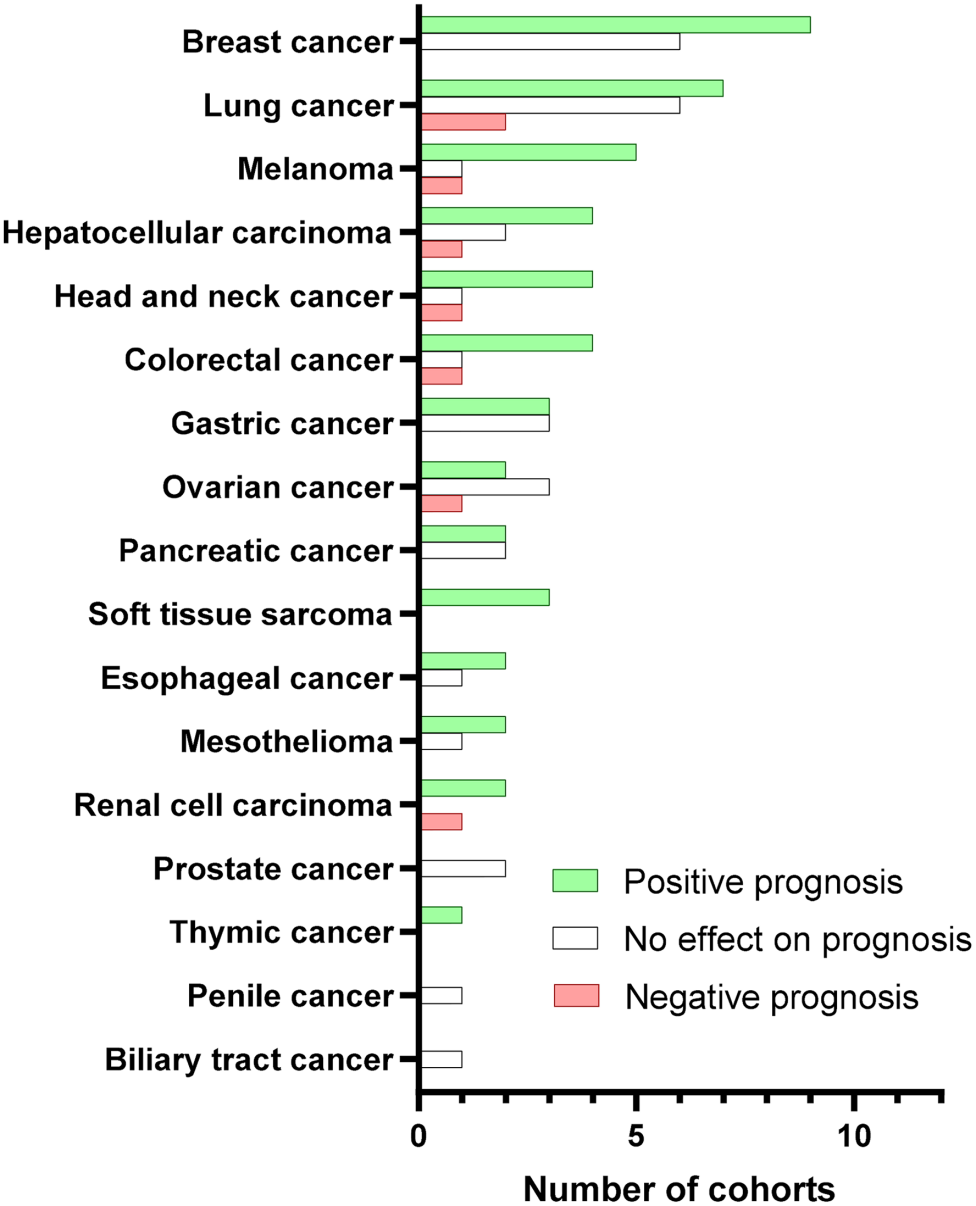
Prognostic value of CD20^+^ TIBs according to cancer type. Bars represent the number of cohorts with positive (green), no effect (white), or negative (red) prognostic significance for the indicated cancer types. We searched PubMed for peer-reviewed articles reporting on the prognostic value in human solid tumors due to the obvious confounding issues. The following search terms and logic gates were used for the PubMed search: “B-cell” AND “cancer” AND “prognosis”.

Collectively, the large majority of the existing studies mainly tested the pan-B-cell marker CD20 in tumor tissues by immunohistochemical staining. More than half of CD20^+^ TIL-B-based studies showed a positive prognostic role of tumoral B cells in cancer patients, whereas less than 10% indicated a negative value (**Figure 2**). Additionally, a meta-analysis of 19 cancer types supported the positive prognostic significance of TIL-Bs in solid tumors (14). Some studies using B-cell subset markers exhibited more conflicting prognostic potential. For example, activated GC-B cells were always a favorable prognostic factor (36), whereas regulatory B cells (Bregs) mostly acted as an unfavorable biomarker (52). Moreover, the prognostic influence of B cells was generally stronger when T cells (41) or TLSs (6–8) were co-localized with B cells in tumors. Therefore, it is advisable that more biomarkers are adopted to distinguish the prognostic value of different B-cell subpopulations as well as their co-existence with TLSs or other immune components in the future.

## Antitumor immunity of B cells

B cells can have bipolar properties, either tumor suppression or tumor promotion, depending on their subtypes and the complicated dynamic TME. TIL-Bs have been reported to exert antitumor functions *via* a direct tumor-killing effect, antigen presentation, antibody production, cytokine secretion, and other activities (**Figure 1**).

### Tumor-killing B cells

Adoptive cellular therapy (ACT) based on T cells has been extensively studied (53). However, the ideal strategy should appropriately stimulate both cellular immunity and humoral immunity. Indeed, activated B cells also have the potential to kill tumor cells *via* antibody-dependent and -independent mechanisms (**Figure 1**) (54–59), which provides the possibility for ACT based on B cells. Our laboratory previously reported the tumor-killing abilities of B cells primed *in vivo* and activated *in vitro* (56–59). These activated B cells were adoptively transferred into tumor-implanted mice, leading to significant tumor regression in primary subcutaneous tumors as well as metastatic pulmonary tumors (56). This phenomenon has been confirmed in three tumor histologies, including breast cancer, melanoma, and sarcoma (56–59). In addition to producing tumor-specific antibodies that killed tumor cells *via* complement-dependent cytotoxicity, activated B cells harvested from tumor-draining lymph nodes (TDLN) were able to induce tumor-cell lysis in an antibody-independent way (58, 59). Of note, combined adoptive transfers of activated TDLN B cells and T cells resulted in stronger antitumor responses than the transfer of either cell population alone (56, 57).

The potential mechanisms of B cell-mediated direct killing effect appear to be multiple. Tao *et al.* found that effector B cells can directly kill tumor cells *via* the Fas/FasL pathway, and was inhibited by IL-10 (58). In that study, IL-10^-/-^ B cells mediated a stronger antitumor effect compared to wild-type B cells, which was verified by IL-10 neutralization utilizing antibody (58). Furthermore, B cell killing of tumor cells was impaired by anti-FasL antibody in a dose-dependent manner (58). In a separate study, Xia et al. defined additional mechanisms involved in cancer ACT based on activated TDLN B cells (59). In that study, IL-2 significantly amplified the therapeutic activity of transferred effector B cells (59). Additional *in vitro* experiments demonstrated that activated B cells provoked direct cytotoxic action on tumor cells through CXCR4/CXCL12 pathways, while without cell contact, B cell-secreted perforin also led to tumor cell cytotoxicity (59).

Additional publications support the fact that B cells can act as effector cells with tumor-killing capacity. Kemp et al. demonstrated that the stimulation of human peripheral blood mononuclear cells with CpG-A oligodeoxynucleotide (ODN) led to high levels of functional B cells, which mediated tumor cell lysis *via* inducing expressions of functional TRAIL/Apo-2 ligand on B cells (60). Penafuerte and colleagues reported that activation of B cells with a chimeric protein consisting of the ectodomain of TGF-β receptor II and IL-2 resulted in effector B cells that induced potent antitumor immunity (61). In addition, it was found that CD73 inhibition elicited sustained B cell-mediated tumor regression in melanoma-bearing mice (62). Together, these studies suggested that with multiple cellular and molecular events, B effector cells can mediate therapeutic antitumor function *via* direct killing of the tumor cells.

### The role of B cells as antigen-presenting cells

B cells may function as antigen-presenting cells (APCs) to engage with T cells *in situ*, and thereby trigger anti-cancer responses (**Figure 1**). It has been reported that human B cells can efficiently present peptides to CD4^+^ T cells after being activated by CD40 ligand and pulsed with tumor antigen (63). In agreement with this finding, we have reported that activated B cells administered in the ACT will confer host T cell immunity *via* its antigen-presenting function utilizing a murine congenic model where donor *vs.* host cells was tracked (57). Intriguingly, Colluru and colleagues successfully applied B cells as a tool to present tumor DNA to CD8^+^ T cells as a vaccine, which elicited an antitumor effect *in vivo* (64). In another report, B cells were reported to infiltrate into the brain tumor site, where they promoted T cell-induced tumor killing as APCs (65).

Bruno et al. observed that TIL-Bs isolated from human NSCLC tumors efficiently presented antigens to CD4^+^ TILs (33). Interestingly, two subgroups of TIL-Bs with opposite functions were described as activated (CD19^+^CD69^+^CD27^+^CD21^+^) or exhausted (CD19^+^CD69^+^CD27^−^CD21^−^) TIL-Bs (33), which induced Th1 phenotype CD4^+^IFN-γ^+^ or Treg phenotype CD4^+^Foxp3^+^ cells, respectively (33). In addition, B cells from doxorubicin-treated cancer patients were found to have enhanced expression of CD86 with improved APC ability (66).

A recent study reported by Jiang et al. demonstrated that CD19^+^ TIL-Bs expressing APC-characteristic markers were found to be co-localized with activated CD4^+^ TIL-Ts in human bladder cancer and was associated with improved survival (67). Using an antigen-presentation assay in this study, TIL-Bs markedly promoted proliferation and activation of CD4^+^ TIL-Ts, which was completely abrogated by HLA-DR blockade (67). In breast tumor-bearing mice, ICB-treated tumors presented a striking elevation in APC B cells (10). When B cells were depleted by anti-CD20 or anti-CD19 antibodies, the effector memory and central memory subsets of CD4^+^ T cells, as well as the effector and effector memory subsets of CD8^+^ T cells, were remarkably decreased (10). More recently, the abundance and spatial distribution of CD86^+^ antigen-presenting B cells have been described in tumor samples of cancer patients, indicating that they are elevated in TILs, localized in TLS, and enriched in tumors with increased TLSs (68). *In vitro* study further revealed that the specific B cell subpopulation can trigger T cell-mediated immune responses, confirming that they are professional APCs in tumor immunity (68).

Taken together, these findings suggested that TIL-Bs can serve as APCs to mediate antitumor responses, providing a better chance of B cells in developing additional immunotherapeutic strategies.

### Tumor-specific antibody-producing B cells

Multiple lines of evidence have demonstrated that B cells act as an antitumor player *via* antibody-dependent cytotoxicity to tumor cells. Reports have established that B cells within tumor-related TLSs can convert to antibody-producing memory B cells or plasma cells after exposure to tumor-associated antigens (**Figure 1**) (19, 69). Thus, TIL-Bs usually serve as an efficient source of tumor-related antibodies, which can be tested in the serum and tumor tissue from cancer patients (69–71). Importantly, detection of serum autoantibodies has been proposed as early clues for human malignancies (72). Additionally, increased tumor-infiltrating plasma cells have been reported to be correlated with better prognosis in ovarian cancer (73), breast cancer (74), colorectal cancer (74), NSCLC (74), and gastric cancer (75), which further support the antitumor property of tumor-targeting antibodies.

Indeed, tumor-infiltrating plasma cells are able to produce oligoclonal, somatically mutated antibodies with the capacity to bind tumoral antigens, provoking the specific cytotoxicity to tumor cells. Our preclinical studies showed that immunoglobulin G (IgG) harvested from tumor-bearing mice bound selectively to tumor cells, triggering tumor cell lysis *via* complement-dependent cytotoxicity (CDC) (57–59). More recently, we further demonstrated that the immunotherapies targeting integrin β4 induced host immunity against cancer stem cells *via* specific CDC (76). In a separate study, Carmi et al. revealed that allogeneic tumor rejection was mediated in the murine model at the early stage of tumor development by naturally occurring tumor-specific IgG antibodies, which prompted DCs to internalize tumor antigens and subsequently activate T cells, initiating a cytotoxic effect to control tumor growth (77). More recently, Hollern reported that tumor-associated IgG was essential in mediating the response to ICBs (10). In that study, ICB-induced B cell activation resulted in the generation of class-switched plasma cells and increased tumor-specific serum IgG (10). Both the loss of antibody secretion and neutralization of FcR during ICB therapy diminished the initial antitumor response in breast tumor-bearing mice (10). This work for the first time identified a crucial role for antibody secretion in the function of ICB therapy.

Very recent studies have shown that intratumoral B cell-generated autoantibodies induce tumor cell apoptosis *via* targeting proteins on the surface of tumor cells (12, 13). Mazor et al. have reported that tumor cells derived from patients of high-grade serous ovarian carcinoma were frequently coated with IgGs produced by intratumoral antibody-secreting cells (12). These tumor-reactive antibodies can target MMP14 on the surface of tumor cells and induce NK cell-mediated antibody-dependent cellular cytotoxicity (ADCC) (12). Another study has also shown that high levels of IgG-producing plasma cells and IgG-coated and apoptotic tumor cells coexist in TLS^+^ tumors, suggesting antibody-mediated anti-tumor immunity (13). These findings provide the direct proof of tumor-killing activity of autoantibodies generated by B/plasma cells.

Collectively, these findings strengthened the premise that humoral responses can have potent antitumor activities *via* tumor-specific antibody-facilitated tumor cell lysis.

## Tumor-promoting effects of Bregs and antibodies

Intratumoral B cells and antibodies may also act as immunosuppressive players, thereby driving tumor growth. B cells with protumor properties are mostly described as Bregs, which have been identified by cell surface markers and secretion of cytokines, functioning by suppressing immune responses that prompt tumor progression (**Figure 1**). Clinical cohort studies report that Breg enhancement in the TME is correlated with Tregs, predicting poorer outcomes in cancer patients (52, 78, 79), which suggests that Bregs might actively participate in tumor immune escape and cancer progression.

### Tumor-promoting functions of Bregs

Bregs, first reported by Morris (80), are special subsets of B cells with immune-suppressing properties for maintaining immune tolerance. Recently, a new wave of studies has emerged on tumor-infiltrating Bregs, which appears to be disadvantageous to cancer patients’ outcomes. In breast cancer, the co-localization of Bregs and Tregs was associated with shorter metastasis-free survival (52). Additionally, high infiltration of programmed cell death-1 (PD-1)^hi^ Bregs presenting in advanced-stage HCC, correlated with early recurrence (78). Similarly, programmed death-ligand 1 (PD-L1)^+^ Bregs were found to be enriched in human metastatic melanoma tumors compared to primary lesions (79).

Within various secondary lymphoid tissues, naïve B cells are activated once exposed to antigens, and then proliferate and form GCs, where they receive signals to grow and differentiate into memory B cells and plasma cells. As a subpopulation of B cells, Bregs have been shown to arise at multiple stages during B cell development (81). Typically, the phenotypes in human tumors are enriched within the CD38^+^ transitional (78, 82–85) and CD27^+^ memory B cell population (**Table 1**) (86, 88, 90). In addition, a novel type of Bregs identified by Shalapour et al. share the phenotypes with IgA^+^CD138^+^ plasma cells (**Figure 1**) (91). Meanwhile, the precursors of several Bregs (e.g., adenosine-producing B cells) remained to be clarified (92). Also, some Bregs were characterized by functional molecules, such as PD-1 (78), PD-L1 (79, 88), T-cell immunoglobulin and mucin domain-1 (TIM-1) (83, 93), granzyme B (85), and CD5 (89). Together, it is thought that all B cell subpopulations can obtain an inhibitory property with appropriate stimuli (81), making their descriptions even harder due to multiple subtypes.

**Table 1:** Breg phenotypes in human solid tumor.

Breg subsets	Phenotype(s)	Immunosuppressive functions or features	Cancer types	References
IL-10-producing B cells	CD19^+^CD24^hi^CD38^hi^	Suppression of IFN-γ and TNF-α by CD4^+^Th cells, and introduction of CD4^+^FoxP3^+^ Tregs *via* TGF-β1	Gastric cancer	(84)
	CD19^+^ CD27^+^ CD10^-^	Production of IL-10, alteration of the cytokine production profile by CD4 and CD8^+^ T cells	Gastric cancer	(86)
	CD19^+^CD25^+^	Production of IL-10, and correlation to introduction of Tregs	Breast cancer	(52)
IL-35-producing B cells	CD19^+^CD24^hi^CD38^hi^	Production of IL-35 and IL-10, introduction of pSTAT3^+^CXCR3^-^CD8^+^ T cells	Pancreatic cancer	(82)
	CD19^+^CD1d^+^CD5^+^	Production of IL-35, and promotion of tumor growth	Pancreatic cancer	(87)
TGF-β-producing B cells	CD19^+^CD24^hi^CD38^hi^	Suppression of IFN-γ and TNF-α by CD4^+^Th cells, and introduction of CD4^+^FoxP3^+^ Tregs *via* TGF-β1	Gastric cancer	(84)
PD-1^hi^ B cells	CD5^+^CD24^-/+^ CD27^hi/+^CD38^dim^	Suppression of tumor-specific T cells and promotion of tumor growth *via* IL-10 signals	Hepatocellular carcinoma	(78)
PD-L1^+^ B cells	CD20^+^CD27^-^IgM^hi^IgD^hi^	Inhibition of IFN-γ by CD4^+^ and CD8^+^ T cells	Melanoma	(79)
	CD19^+^CD24^+^CD38^+^	Positive correlation with Tregs and negative association with PD-1^hi^ effector cells	Breast cancer	(88)
TIM-1^+^ B cells	CD5^hi^CD24^-^CD27^-/+^CD38^+/hi^	Production of IL-10, and suppression of CD8^+^ T cell activity	Hepatocellular carcinoma	(83)
CD5^+^ B cells	CD19^+^CD5^+^	Induction of angiogenesis and inhibition of IFN-γ secretion	Prostate cancer, lung cancer	(89)
Plasmablasts	CD19^lo^CD27^hi^	Production of IL-10 and inhibition of IL-17A expression	Colorectal cancer	(90)
IgA^+^ cells	IgA^+^B220^-^CD138^-/+^	Expression of PD-L1 and IL-10, and direct suppression of liver cytotoxic CD8^+^ T cells	Hepatocellular carcinoma	(91)
Granzyme B^+^ cells	CD19^+^CD38^+^ CD1d^+^IgM^+^CD147^+^	Production of IL-10 and inhibition of T cell proliferation	Breast, ovarian, cervical, colorectal, and prostate cancer	(85)
Adenosine-producing B cells	CD19^+^CD37^+^	Production of adenosine and inhibition of Bruton’s tyrosine kinase and Ca^2+^ influx in B effector cells	Head and neck cancer	(92)

Despite the confusing distinct phenotypes, Bregs can be functionally defined by their capacities for the generation of immunosuppressive cytokines, including IL-10 (86), IL-35 (82), and TGF-β (**Figure 1**) (84). For example, a protumorigenic PD-1^hi^ Breg subset in human HCC exhibited a unique CD5^hi^CD24^−/+^CD27^hi/+^CD38^dim^ phenotype, which caused T-cell dysfunction and fostered tumor progression *via* IL-10-dependent pathways (78). In another study, a granzyme B^+^ Bregs with a CD19^+^CD38^+^CD1d^+^IgM^+^CD147^+^ expression signature was found in various human solid tumors (85). They can be induced by IL-21 and generate high amounts of IL-10 (**Figure 1**) (85). Similarly in HCC, the intratumoral TIM-1^+^ Bregs with a CD5^hi^CD24^-^CD27^-/+^CD38^+/hi^ phenotype generated high levels of IL-10 and exerted robust repressive functions against CD8^+^ T cells (83). Another type of IL-10-secreting Bregs (B10) was characterized by a CD19^+^CD24^hi^CD38^hi^ phenotype in gastric cancer (84). Besides, IL-35-producing CD1d^hi^CD5^+^ Bregs accumulated in pancreatic cancer and supported tumor cell growth, underlining the potential value of IL-35 as a therapeutic target (87).

Several studies report that Bregs not only directly fostered tumor growth, but also acted on other immune compartments in the TME. Mostly, Bregs negatively regulate the antitumor immune responses of T cells. Among different types of Bregs, B10 has been shown to suppress the secretion of proinflammatory cytokines by CD4^+^ Th cells and induce CD4^+^FoxP3^+^ Tregs (52, 84, 86). Furthermore, an *in vitro* study demonstrated that B10 inhibited the proliferation and activation of T cells (52, 84, 86). IL-35-producing Bregs induced pSTAT3^+^CXCR3^-^CD8^+^ T cells in pancreatic cancer (87). Additionally, Bregs promote tumor progression *via* interplay with myeloid-derived suppressor cells, tumor-associated macrophages, and natural killer cells (**Figure 1**).

However, the immunosuppressive characteristics and mechanisms of tumor-infiltrating Bregs are still largely unclear, which remains a central focus of investigation.

### Tumor-promoting effects of antibodies

In addition to Bregs, local or systemic tumor-associated autoantibodies may also exert a protumoral effect through initiation and maintenance of inflammation (91), tissue remodeling (94), and angiogenesis (94). Clinical cohort studies exhibited a close association of serum p53 antibody with poor prognosis in breast cancer patients (95). Other serum antibodies were also reported to correlate with poor survival in ovarian and pancreatic cancer (96). Generally, elevated immunoglobulins (97) and high IgG3-1 switches (98) have been identified as unfavorable prognostic biomarkers in renal cell carcinoma. These data from a clinical perspective suggest that some tumor-specific antibodies might promote tumor processes.

It is important to note that antibody functionality, protumor or antitumor, may be determined by the antibody isotype. Remarkably, a study using TCGA RNA-seq data indicated the association of high fractions of IgA, IgD, or IgE with a poor prognosis in human melanoma (99). A low proportion of intratumorally produced IgA was specifically correlated with improved overall survival for KRAS mutation lung adenocarcinoma (71). Karagiannis et al. reported that antigen-specific and nonspecific IgG4 hampered IgG1-induced antitumor activities through the deactivation of FcγRI, which explained the inverse correlation between serum IgG4 and patient survival (100). Additionally, RNA-seq data have shown that the relative proportions of IgG3, in contrast to IgG1, was either neutrally or negatively associated with outcome in melanoma (99).

In summary, existing evidence indicates that some phenotypes of immunoglobulin are associated with worse outcomes in cancer patients. Animal studies also illustrate that tumor-specific antibodies can promote tumor development by dismantling antitumor immunity. However, the mechanisms underlying antibody-mediated protumor properties have not yet been fully investigated. More work needs to be conducted in this field to elucidate the obstacles to cancer immunotherapy.

## Strategies of cancer immunotherapies involved with B cells

Considering the bidirectional roles of B cells in tumor initiation and development, targeting B cells could be promising in cancer immunotherapy. In light of both antitumor and protumor properties of B cells driven by distinct subpopulations and the complexity of tumor context, precise therapies targeting specific B cells could lead to more favorable therapeutic outcomes.

### Therapeutic strategies based on antitumor responses of B cells

Previous studies in our laboratory and others have demonstrated that activated B cells can specifically and directly kill tumor cells (16, 58, 59), supporting the potential of adoptive B cell therapies. It has been well established that adoptive T cell therapy, such as CAR-T cell therapy (2), is capable of inducing high clinical response rates in hematological malignant disorders (53), whereas attempts to treat patients with solid tumors using CAR-T cells are still in their infancy. Preclinical studies have shown that the co-transfer of activated T cells and B cells led to a greater extent of tumor regression than either cell population alone (56, 57), suggesting effector B cells can function as a valuable adjunct in T cell-based immunotherapy. Moreover, adoptive transfers of other types of B cells, like TIM-4^+^ B cells (101), GIFT4-programmed B cells (102), and CpG-ODN activated B cells (103), also have capacities to elicit tumor regression. These studies offer novel cancer immunotherapeutic approaches based on B cells.

Resting B cells with a poor capacity of antigen presentation can be activated by diverse stimuli to obtain immunostimulatory ability. CD40 signals are the strongest inducer of antigen-presenting B cells to trigger potent T-cell immunity (104). CD40-activated B (CD40-B) cells are thought to be an encouraging alternative to DCs as skilled APCs for antigen-specific cancer immunotherapy (105). In contrast to DCs, a greater number of B cells can be easily harvested from human peripheral blood after *in vitro* expansion in the presence of CD40L, without the loss of APC functionality (106). CD40 signal activation markedly enhances the antigen-presenting ability of B cells, powerfully inducing vigorous expansion of antigen-specific T cells and homing to the secondary lymphoid organs (55, 105). Importantly, tumor-implanted mice were preventively immunized by tumor antigen-primed CD40-B cells, resulting in a protective antitumor response against melanoma and lymphoma (105). In summary, CD40-B cells emerge as an attractive tool to develop additional immunotherapeutic agents. The design of clinical trials using CD40-B cells to confirm this potential antitumor function seems worthy.

Effector B cells also can be induced or activated by cytokines or chemotherapy drugs, which can be utilized to enhance B-cell-mediated antitumor responses. Li and colleagues found that IL-2 augmented the antitumor responses of adoptively transferred TDLN B cells (59). A separate study reported that IL-17A improved the tumor-killing activity of B cells in esophageal squamous cancer (16). In addition, Lu et al. demonstrated that chemotherapy in breast cancer patients induced a novel ICOSL^+^ B cell subset with antitumor capacity by enhancing the effector T cells to Tregs ratio (9). Similarly, doxorubicin can induce CD86 expression on the surface of B cells to elevate their antigen-presenting capacity in urinary bladder cancer patients (66). Thus, conventional drugs, as well as cytokines, could be a favorable option utilized to induce effector B cells and improve the antitumor efficacy of immunotherapy involved with B cells.

### Therapeutic strategies targeting protumor functions of B cells

Since several lines of evidence indicate that some subpopulations of B cells possess tumor-promoting activities, the anti-CD20 antibody (rituximab) has been used to deplete the total B cell population for the treatment of solid tumors in small cohorts of patients with melanoma (107) and advanced colon cancer (108). Although it was difficult to estimate the therapeutic value from such small cohorts of patients, it seemed that B cell-depletion therapy was not associated with significant toxicity (107, 108). In another study, rituximab therapy combined with low-dose IL-2 in patients with melanoma and renal cell carcinoma did not show any clear benefit of the B cell depletion (109). Of note, a recent study demonstrated that B-cell depleting or inhibiting treatment in mammary tumor-bearing mice significantly weakened the antitumor immunity of combined ICB immunotherapy (10). In addition, most of the cohort studies revealed the positive association of TIL-Bs with better outcomes in various solid tumors (14). Therefore, more precise immunotherapies targeting specific B cell subsets (e.g., Bregs) instead of the total B cell population would be more rational for further investigations.

Indeed, strategies targeting Bregs have been reported to be effective in murine tumor models. For example, Li et al. demonstrated that IL-10 deletion or blockade notably improved the therapeutic effect of TDLN B cells in an ACT model (58). Additionally, a recombinant plasmid pcCD19scFv-IL10R containing the gene of the extracellular domain of IL-10R1 and anti-CD19 single-chain variable fragment significantly decreased B10 cells and Tregs, which may offer a new immunotherapeutic strategy targeting Bregs (110). Interestingly, both conventional chemotherapy (66) and novel molecular targeted therapy (111) were reported to serve as Breg inhibitors *via* the downregulation of IL-10 secretion. IL-35, another critical immunosuppressing factor derived from Bregs, has been proved to be a potential immunotherapy target. Mirlekar reported that specific deletion of IL-35 in B cells overcame immunotherapy resistance to anti-PD-1 through activation of CD8+ T cells in murine pancreatic cancer model (80).

In short, B cell-based immunotherapy should consider both antitumor and protumor functions of B cells. Therefore, the basic strategy is to precisely target specific B cell subsets by activating/transferring antitumor effector B cells or suppressing/eliminating protumor inhibitory B cells.

## B cells as biomarkers for therapeutic response

Although B cells have been described as biomarkers of unfavorable outcomes in limited studies, most reports revealed that B cells, especially their co-existence with TLSs or T cells, were associated with improved prognosis of cancer patients (6–8). As a biomarker for the therapy-related response, one study demonstrated that the number of B cells in melanoma tumors was not associated with response to anti-PD-1 therapy or cancer patient survival (112). However, more studies have implied that the presence of enriched TIL-Bs has a strong predictive value in the response to ICB therapy (6–8, 113), suggesting that B cells are not simply bystander cells in the TME, but active participants involving immunotherapies.

Conflicting results of B cell depletion as an adjunct to immunotherapy and chemotherapy also exist. A preclinical study reported that B cell-depletion (anti-CD20) or B cell lack (muMT mice) neither affected tumor growth or animal survival nor improved or hampered the antitumor response to anti-PD-1 therapy (112). In contrast, another report showed that the therapeutic benefit of combined ICB therapies was completely ablated in B cell-inhibited (anti-CD19) and/or B cell-depleted (anti-CD20) tumor-bearing mice (10), establishing the point that B cells may fundamentally orchestrate ICB-driven antitumor responses. In human melanoma, Griss et al. found that TIL-Bs were vital to melanoma inflammation which in turn resulted in CD8^+^ T cell infiltration through the secretion of chemokines (114). In their study, they found that TIL-B was a predictor for survival and response to ICB therapy (114).

Similarly, there exist controversial roles of tumoral B cells in conventional chemotherapy. An early study illustrated that the loss of B cells (anti-CD20) significantly enhanced responses to cisplatin, carboplatin, and paclitaxel through the increase of tumor CD4^+^ and CD8^+^ T cell infiltration (115). In melanoma patients, resistance to BRAF and/or MEK inhibitors was associated with increased levels of intratumoral CD20 transcripts (116). In that study, the use of B cell depletion (rituximab) in chemotherapy-resistant melanoma patients demonstrated antitumor activity, indicating that chemotherapy resistance was mediated by intratumoral B cells (116). Conversely, a recent work elucidated that a novel ICOSL^+^ B cell subset in breast cancer tissues emerging after chemotherapy boosted the chemosensitivity by increasing tumor-specific CD8^+^ T cells and the Th1/Treg ratio, supporting that the effector B cell subtype exerted antitumor immunity to reverse the chemoresistance (9).

Considering the fact that tumor-promoting and -suppressing B cells simultaneously exist in the TME, the one-dimensional intervention of total B-cell depletion may remove effector B cells and/or enrich Bregs, thereby producing a harmful therapeutic effect on cancer patients. Thus, total B cell depletion should be chosen carefully and only applied to those patients whose TIL-Bs are verified to be definitely and completely immunosuppressive.

## Concluding remarks

In this review, we have summarized some key issues involved with B cells in tumor immunity. Over the years, studies have painted an ambiguous role of B cells in tumor immunology, which might be explained by their phenotypic and functional heterogeneity. All the above contradictory findings concerning B cells in the TME, including the field of cancer prognosis and treatment, may originate from the spatial and longitudinal heterogeneity of B cells. Therefore, there is an urgent need to dissect the mechanisms responsible for B cell heterogeneity in tumors. It would largely help design new clinical strategies for B cell-based immunotherapy with further characterization and description of B cell subsets in tumor immunity.

## Author contributions

QL, AC, and YQ developed the concept. YQ and FL reviewed the literature and wrote the review. QL, AC, and KL provided invaluable editing and review of the manuscript. All authors contributed to the article and approved the submitted version.

## Funding

This work was partially supported by Gillson Longenbaugh Foundation (AC and QL) and Cancer Center, Union Hospital, Tongji Medical College, Huazhong University of Science and Technology (YQ).

## Conflict of interest

The authors declare that the research was conducted in the absence of any commercial or financial relationships that could be construed as a potential conflict of interest.

## Publisher’s note

All claims expressed in this article are solely those of the authors and do not necessarily represent those of their affiliated organizations, or those of the publisher, the editors and the reviewers. Any product that may be evaluated in this article, or claim that may be made by its manufacturer, is not guaranteed or endorsed by the publisher.

## Abbreviations

ICB,1 immune checkpoint blockade; CAR-T, chimeric antigen receptor-T; TIL-Bs, tumor-infiltrating B lymphocytes; TME, tumor microenvironment; TILs, tumor-infiltrating lymphocytes; HEVs, endothelial venules; TLSs, tertiary lymphoid structures; TIL-Ts, tumor-infiltrating T cells; GCs, germinal centers; fDCs, follicular dendritic cells; DCs, dendritic cells; NSCLC, non-small-cell lung cancer; Treg, regulatory T cell; Bregs, regulatory B cells; ACT, adoptive cellular therapy; TDLN, tumor-draining lymph node; ODN, oligodeoxynucleotide; APCs, antigen-presenting cells; Ig, immunoglobulin; PD-1, programmed cell death-1; HCC, hepatocellular carcinoma; PD-L1, programmed death-ligand 1; TIM, T-cell immunoglobulin and mucin domain; B10, IL-10-secreting Bregs; CD40-B, CD40-activated B.
